# Supplementary material for: Genome-Wide Identification, Evolution, and Expression Analysis of TPS and TPP Gene Families in Brachypodium distachyon
Source: Plants (Basel). 2019 Sep 23;8(10):362. doi: 10.3390/plants8100362 (PMC6843561; doi:10.3390/plants8100362)
Supplement: Supplementary file 1 [file plants-08-00362-s001.zip › Table S1.docx]

**Table S1** The primer sequences used in this work

| **Name** | **Sequence** |
| --- | --- |
| qBdTPS1-F | GAAATTGTTGGGCGCATAAATG |
| qBdTPS1-R | GTAGCTTACAAGATTCATGCCG |
| qBdTPS2-F | GAGTGGTGCTATTCGTGTAAAC |
| qBdTPS2-R | TGCTCACATACTTGTAGTGCTT |
| qBdTPS3-F | CTGATTTCGTGCTCTGTATTGG |
| qBdTPS3-R | CTGCAGCATCTTAATGACGTC |
| qBdTPS4-F | CTTCTACAAATTGGCTTCTCGG |
| qBdTPS4-R | CTCCACAGCTCATCTCTAGAAG |
| qBdTPS5-F | AAACACCTCTGTTTGCATGTAC |
| qBdTPS5-R | CTAGTGAAGAGACTGACGTAGC |
| qBdTPS6-F | GAGAGCAGGACAATATTGCTTG |
| qBdTPS6-R | CAAAACTTCTGCACTAGGTGTC |
| qBdTPS7-F | GATCCCCAAACCTTGTTGTATG |
| qBdTPS7-R | GTATCTTCGATTCTTCCGGTGA |
| qBdTPS8-F | GGATACTGGGCTAATAGCTTCC |
| qBdTPS8-R | CTGTTCAGTATCTGGACCGATT |
| qBdTPS9-F | AATGCAGATTTGATTGGGTTCC |
| qBdTPS9-R | AACAGTCCGTCCATAATACTCC |
| qBdTPPA-F | TGACGTACACAACACCTACTAG |
| qBdTPPA-R | AGATCGTCAGCTTGAATATCGT |
| qBdTPPB-F | ATAAGATTGGAACCATTGCGTG |
| qBdTPPB-R | GGGTGCCATCATAGTCTAAGAA |
| qBdTPPC-F | CTTCGAACATGATGCCGTTTTC |
| qBdTPPC-R | AATTCTCCTGGCCAAAGACTAA |
| qBdTPPD-F | CGAAAGGTTTTAGAGGTTCGTC |
| qBdTPPD-R | ACCAGTATTCCAAATCCACAGT |
| qBdTPPE-F | CCTCGAAGAAGAGAGTAGTTCC |
| qBdTPPE-R | GTCATCTCTCTGTCTGACTGTC |
| qBdTPPF-F | CTTCAGATGCGTCGACGAAAAG |
| qBdTPPF-R | CACCTCCAACACCATCCTC |
| qBdTPPG-F | GAAGAAGGTCGTCATGTTCCTC |
| qBdTPPG-R | CTCATCGCGTCGGTTATGTAG |
| qBdTPPH-F | CAGAGACAAGGTGTTCAACTTC |
| qBdTPPH-R | AACTTGTTGTTCTCCACCTTGG |
| qBdTPPI-F | CTCTCCGAGCTATACTACGCAG |
| qBdTPPI-R | AACTTGTTGTTCTCCACCCTG |
| qBdTPPJ-F | GATCGTGATGTTCCTCGACTAC |
| qBdTPPJ-R | TCACTGCGTCTCTCATATCTTC |
| BdSamDC-F | TGCTAATCTGCTCCAATGGC |
| BdSamDC-R | GACGCAGCTGACCACCTAGA |
