# Supplementary material for: Genome-Wide Identification, Evolution, and Expression Analysis of TPS and TPP Gene Families in Brachypodium distachyon
Source: Plants (Basel). 2019 Sep 23;8(10):362. doi: 10.3390/plants8100362 (PMC6843561; doi:10.3390/plants8100362)
Supplement: Supplementary file 1 [file plants-08-00362-s001.zip › Table S2.docx]

**Table S2** The predicted interaction transcription factor and GO analysis

| **Gene** | **Transcription factor** | **Family** | **Biological Process** |
| --- | --- | --- | --- |
| Bradi2g19640 | Bradi1g20586 | MYB | Regulation of transcription, DNA-templated; embryo sac development; guard cell differentiation |
|  | Bradi2g37130 | Dof | Photomorphogenesis; positive regulation of transcription, DNA-templated |
|  | Bradi3g32090 | MIKC_MADS | Protein import into nucleus, translocation; response to cold and gibberellin; positive regulation of flower development and transcription, DNA-templated; maintenance of inflorescence meristem identity |
|  | Bradi3g39927 | bHLH | Photoperiodism; flowering |
|  | Bradi4g23270 | B3 | Regulation of transcription, DNA-templated; response to cold; cellular protein localization |
| Bradi3g37200  Bradi3g53790  Bradi3g35820  Bradi2g19710  Bradi2g49870  Bradi4g41580  Bradi1g69420  Bradi4g29730  Bradi3g32970  Bradi1g27470  Bradi3g50810  Bradi5g17890  Bradi3g35590  Bradi4g29030  Bradi3g58960  Bradi1g60950  Bradi1g45480  Bradi1g21420 | Bradi4g23270  Bradi4g33770  [Bradi2g01960](http://planttfdb.cbi.pku.edu.cn/tf.php?sp=Bdi&did=Bradi2g01960.2.p)  Bradi2g06330  Bradi2g24940  Bradi2g37130  Bradi3g32090  Bradi3g52880  Bradi4g23270  Bradi5g26110  [Bradi1g48320](http://planttfdb.cbi.pku.edu.cn/tf.php?sp=Bdi&did=Bradi1g48320.1.p)  Bradi2g11100  Bradi2g57201  Bradi2g58130  Bradi3g38200  Bradi3g51800  Bradi3g54160  Bradi4g23270  Bradi4g39630  Bradi5g17610  Bradi1g63170  Bradi2g05760  Bradi2g23710  Bradi2g36730  Bradi4g24937  Bradi4g35240  Bradi5g26110  Bradi1g00666  Bradi1g18580  Bradi1g45220  Bradi1g46690  Bradi2g08480  Bradi2g21067  Bradi2g27920  Bradi2g29960  Bradi2g37130  Bradi2g49860  Bradi2g08460  Bradi1g77217  Bradi2g05760  Bradi2g23710  Bradi3g07540  Bradi3g26910  Bradi3g33400  Bradi4g33740  Bradi4g34157  Bradi1g13980  Bradi1g23550  Bradi1g38180  Bradi1g48400  Bradi2g42240  Bradi3g02800  Bradi3g59380  Bradi4g21265  Bradi1g49980  Bradi1g77217  Bradi2g50540  Bradi4g33750  Bradi4g34022  Bradi4g42050  Bradi1g10047  Bradi1g18580  Bradi1g72150  Bradi2g07357  Bradi2g21067  Bradi2g37130  Bradi4g21265  Bradi4g27850  Bradi4g37147  Bradi4g38941  Bradi1g34470  Bradi2g48057  Bradi5g13041  Bradi1g00666  Bradi1g18580  Bradi1g23756  Bradi1g27170  Bradi1g45220  Bradi1g46690  Bradi1g72990  Bradi2g04270  Bradi2g07357  Bradi1g14750  Bradi3g60352  Bradi4g32967  Bradi1g55710  Bradi2g08460  Bradi2g37130  Bradi2g41550  Bradi3g30457  Bradi3g32090  Bradi3g36370  Bradi3g49987  Bradi3g52880  Bradi3g58770  Bradi1g03020  Bradi1g35920  Bradi2g37130  Bradi2g55980  Bradi2g57100  Bradi3g30457  Bradi3g32090  Bradi3g52880  Bradi4g23270  Bradi2g48057  Bradi1g08340  Bradi1g48525  Bradi1g49980  Bradi2g15940  Bradi2g46190  Bradi2g54640  Bradi3g02980  Bradi3g22040  Bradi3g60018  Bradi4g02570 | B3  SBP  MYB  MIKC_MADS  MIKC_MADS  Dof  MIKC_MADS  Dof  B3  MYB  ERF  bHLH  ERF  C2H2  bZIP  MIKC_MADS  ERF  B3  bZIP  ERF  bZIP  MYB  MYB  MYB  bZIP  bZIP  MYB  ERF  ERF  TCP  ERF  LBD  ERF  ERF  ERF  Dof  HSF  C2H2  NAC  MYB  MYB  bZIP  C2H2  MYB  bZIP  NAC  bHLH  BES1  BES1  bHLH  MYB  BES1  NAC  ERF  BBR-BPC  NAC  CPP  EIL  NAC  E2F/DP  TALE  ERF  MIKC_MADS  ERF  ERF  Dof  ERF  ERF  Nin-like  ERF  G2-like  B3  MYB  ERF  ERF  ERF  CAMTA  TCP  ERF  ERF  LBD  ERF  C2H2  TCP  bHLH  CPP  C2H2  Dof  C2H2  Trihelix  MIKC_MADS  MYB  MYB  Dof  MYB  GATA  C3H  Dof  Dof  NAC  Trihelix  MIKC_MADS  Dof  B3  B3  MIKC_MADS  MYB  BBR-BPC  bZIP  ARF  MYB  bZIP  bZIP  ERF  bZIP | Regulation of transcription, DNA-templated; response to cold;cellular protein localization  Vegetative to reproductive phase transition of meristem; anther development; regulation of leaf formation  Gibberellin biosynthetic process; response to salicylic acid; cellular response to phosphate starvation  Regulation of transcription, DNA-templated; leaf development; carpel development; stamen development; maintenance of floral organ identity  Regulation of transcription, DNA-templated; specification of floral organ identity  Photomorphogenesis; positive regulation of transcription, DNA-templated  Protein import into nucleus, translocation; response to cold; response to gibberellin; positive regulation of flower development; maintenance of inflorescence meristem identity; positive regulation of transcription, DNA-templated  Regulation of transcription, DNA-templated; response to cold; cellular protein localization  Regulation of transcription, DNA-templated; response to cold; cellular protein localization  Regulation of transcription, DNA-templated  Regulation of transcription, DNA-templated  De-etiolation; gibberellic acid mediated signaling pathway; red or far-red light signaling pathway;positive regulation of anthocyanin metabolic process; regulation of seed growth  Regulation of transcription, DNA-templated; response to chitin  Response to brassinosteroid; unidimensional cell growth; vegetative to reproductive phase transition of meristem; histone H3-K9 demethylation; negative regulation of histone acetylation; positive regulation of growth rate; positive regulation of gene expression, epigenetic; leaf development  Response to water deprivation; response to salt stress; abscisic acid-activated signaling pathway; glucose mediated signaling pathway; positive regulation of transcription, DNA-templated  Regulation of transcription, DNA-templated;  Regulation of transcription, DNA-templated  Regulation of transcription, DNA-templated; response to cold; cellular protein localization  Positive regulation of transcription, DNA-templated;  Regulation of transcription, DNA-templated  Regulation of transcription, DNA-templated  Chromatin remodeling; regulation of transcription from RNA polymerase II promoter; positive regulation of histone acetylation  Regulation of transcription, DNA-templated  Vasculature development; response to auxin; xylem development; regulation of stomatal movement; seed coat development; root development  Regulation of transcription, DNA-templated; osmosensory signaling pathway; sulfate transport; DNA mediated transformation; thigmotropism; cellular response to sulfate starvation; negative regulation of cell differentiation; nuclear import  Regulation of transcription, DNA-templated  Regulation of transcription, DNA-templated  Regulation of transcription, DNA-templated; defense response; ethylene-activated signaling pathway  Regulation of transcription, DNA-templated; lateral root morphogenesis; bract development; floral meristem growth; floral meristem determinacy  Cell proliferation; response to cytokinin; response to abscisic acid; response to gibberellin; regulation of seed germination; inflorescence development; regulation of defense response  Regulation of transcription, DNA-templated; detection of hypoxia  Unknown  Regulation of transcription, DNA-templated  Cell death; response to ethylene; response to cytokinin; response to jasmonic acid; heat acclimation; positive regulation of transcription, DNA-templated; response to other organism  Response to water deprivation; response to salt stress; response to abscisic acid; heat acclimation; positive regulation of transcription, DNA-templated  Photomorphogenesis; positive regulation of transcription, DNA-templated  Response to reactive oxygen species; response to chitin; positive regulation of transcription, DNA-templated  Unknown  Regulation of transcription, DNA-templated  Chromatin remodeling; regulation of transcription from RNA polymerase II promoter; positive regulation of histone acetylation  Regulation of transcription, DNA-templated  Regulation of transcription, DNA-templated; response to abscisic acid; gibberellic acid mediated signaling pathway; red or far-red light signaling pathway; regulation of photomorphogenesis; response to red light; response to far red light; response to UV-B; positive regulation of anthocyanin metabolic process; positive regulation of circadian rhythm; response to karrikin  Unknown  Negative regulation of transcription from RNA polymerase II promoter; response to salt stress; response to ethylene; response to abscisic acid; response to gibberellin; response to salicylic acid; response to jasmonic acid; positive regulation of cell growth; response to cadmium ion; leaf development; negative regulation of peroxidase activity  Regulation of transcription, DNA-templated  Regulation of transcription, DNA-templated  Regulation of transcription, DNA-templated; heme biosynthetic process; gibberellic acid mediated signaling pathway; negative gravitropism; negative regulation of photomorphogenesis; red light signaling pathway; negative regulation of seed germination; chlorophyll biosynthetic process  Brassinosteroid mediated signaling pathway; defense response to bacterium; negative regulation of transcription, DNA-templated  Regulation of transcription, DNA-templated  Regulation of transcription, DNA-templated; circadian rhythm; response to cold; response to red light; fruit development; negative regulation of seed germination; carpel development  Nucleosome assembly; regulation of transcription from RNA polymerase II promoter; response to salt stress; response to ethylene; response to auxin; response to abscisic acid; response to gibberellin; response to salicylic acid; response to jasmonic acid; telomeric loop formation;response to cadmium ion  Polysaccharide catabolic process; regulation of transcription, DNA-templated; regulation of shoot system development  Regulation of transcription, DNA-templated  Regulation of transcription, DNA-templated  Regulation of transcription, DNA-templated; response to ethylene  Regulation of transcription, DNA-templated  Unknown  Regulation of transcription, DNA-templated; regulation of sulfur metabolic process; cellular response to iron ion  Regulation of transcription, DNA-templated; cellular response to hydrogen peroxide  Regulation of transcription, DNA-templated  Cell fate specification; regulation of transcription, DNA-templated; xylem and phloem pattern formation; xylem development  Regulation of transcription, DNA-templated; lateral root morphogenesis; bract development; floral meristem growth; floral meristem determinacy  Response to temperature stimulus; negative regulation of flower development; maintenance of floral meristem identity; floral meristem determinacy; negative regulation of transcription, DNA-templated;floral whorl development  Regulation of transcription, DNA-templated; transcription factor import into nucleus; leaf development  Regulation of transcription, DNA-templated  Photomorphogenesis; positive regulation of transcription, DNA-templated  Regulation of transcription, DNA-templated  Regulation of transcription, DNA-templated  Unknown  Positive regulation of abscisic acid-activated signaling pathway; positive regulation of cellular defense response; positive regulation of transcription, DNA-templated; stomatal closure  Regulation of transcription, DNA-templated  Regulation of transcription, DNA-templated; positive regulation of cell proliferation; response to auxin; positive regulation of abscisic acid biosynthetic process; somatic embryogenesis; negative regulation of gibberellin biosynthetic process  Trichome morphogenesis; anther wall tapetum development  Regulation of transcription, DNA-templated; defense response; ethylene-activated signaling pathway  Regulation of transcription, DNA-templated; lateral root morphogenesis; bract development; floral meristem growth; floral meristem determinacy  Response to reactive oxygen species; regulation of transcription, DNA-templated; response to ethylene; cellular response to potassium ion; post-embryonic root development  Positive regulation of transcription from RNA polymerase II promoter  Cell proliferation; response to cytokinin; response to abscisic acid; response to gibberellin; regulation of seed germination;inflorescence development; regulation of defense response  Regulation of transcription, DNA-templated; detection of hypoxia  Regulation of transcription, DNA-templated  Organ boundary specification between lateral organs and the meristem  Regulation of transcription, DNA-templated; transcription factor import into nucleus; leaf development  Response to oxidative stress; response to cold; response to water deprivation; response to wounding; response to high light intensity; response to salt stress; response to abscisic acid; photoprotection; response to chitin; photosynthesis;multicellular organism growth; negative regulation of transcription, DNA-templated  Unknown  Transcription, DNA-templated  Unknown  Transcription regulatory region DNA binding; metal ion binding  Photomorphogenesis; positive regulation of transcription, DNA-templated  Nucleic acid binding; metal ion binding  Mucilage biosynthetic process  Response to cold; response to gibberellin; positive regulation of flower development; maintenance of inflorescence meristem identity  Response to abscisic acid  Response to salicylic acid; response to jasmonic acid; regulation of flower development  Procambium histogenesis;phloem or xylem histogenesis;positive regulation of transcription, DNA-templated  Response to auxin; response to abscisic acid; response to salicylic acid  Cell differentiation; positive regulation of transcription from RNA polymerase II promoter  Unknown  Photomorphogenesis; positive regulation of transcription, DNA-templated  Regulation of transcription, DNA-templated  Regulation of transcription, DNA-templated; response to wounding; negative regulation of abscisic acid-activated signaling pathway  Mucilage biosynthetic process  Protein import into nucleus, translocation; response to cold; response to gibberellin; positive regulation of flower development;maintenance of inflorescence meristem identity; positive regulation of transcription, DNA-templated  Procambium histogenesis; phloem or xylem histogenesis; positive regulation of transcription, DNA-templated  Regulation of transcription, DNA-templated; response to cold; cellular protein localization  Regulation of transcription, DNA-templated; positive regulation of cell proliferation; response to auxin; positive regulation of abscisic acid biosynthetic process; somatic embryogenesis; negative regulation of gibberellin biosynthetic process; photoperiodism, flowering  Meristem structural organization; maintenance of floral meristem identity; floral meristem determinacy; positive regulation of transcription, DNA-templated  Pollen tube guidance; regulation of synergid differentiation  Regulation of transcription, DNA-templated; response to ethylene  Regulation of transcription, DNA-templated; response to abscisic acid  Regulation of transcription, DNA-templated; auxin-activated signaling pathway; vegetative phase change; abaxial cell fate specification; floral meristem determinacy  Unknown  Regulation of transcription, DNA-templated; seed germination  Regulation of transcription, DNA-templated; seed germination  Vasculature development; defense response to insect; regulation of transcription, DNA-templated; response to wounding; cell division  Regulation of transcription, DNA-templated; cellular response to abscisic acid stimulus; cellular response to glucose stimulus |
|  |  |  |  |
