# Supplementary material for: Genome-Wide Identification, Evolution, and Expression Analysis of TPS and TPP Gene Families in Brachypodium distachyon
Source: Plants (Basel). 2019 Sep 23;8(10):362. doi: 10.3390/plants8100362 (PMC6843561; doi:10.3390/plants8100362)
Supplement: Supplementary file 1 [file plants-08-00362-s001.zip › Table S3.docx]

**Table S3.** Detail information of direct interaction predicted Network of BdTPS and BdTPP with other genes

| **Name** | **ID** | **Co-expression gene** | **Molecular function** | **Biological process** |
| --- | --- | --- | --- | --- |
| BdTPS1 | Bradi2g19640 | Bradi2g48610 | ATP binding; ATPase activity; nucleotide binding | Transmembrane transport |
|  |  | Bradi5g12810 | Protein-arginine deiminase activity; Agmatine deiminase activity | Putrescine biosynthetic process |
|  |  | Bradi3g48670 | Adenine phosphoribosyltransferase activity | Adenine salvage;nucleoside metabolic process |
|  |  | Bradi2g43557 | Catalytic activity | Gene silencing by RNA |
|  |  | Bradi2g38130 | Tetraspannin | Tetraspanin family integral membrane protein |
| BdTPS2 | Bradi3g37200 | Bradi4g26730 | Zinc ion binding; protein binding | Histone-lysine N-methyltransferase; PHD zinc finger |
|  |  | Bradi5g24300 | Carboxypeptidase activity; serine-type carboxypeptidase activity;peptidase activity; hydrolase activity | Proteolysis involved in cellular protein catabolic process |
|  |  | Bradi4g40700 | Catalytic activity | Catalysis of a biochemical reaction at physiological temperature |
| BdTPS4 | Bradi3g35820 | Bradi1g28110 | Protein kinase activity; ATP binding | Protein phosphorylation |
|  |  | Bradi3g05292 | Protein binding; Leucine rich repeat N-terminal domain | Unknown |
|  |  | Bradi2g45890 | Nucleotide, metal ion and ATP binding; ribokinase, kinase, transferase and phosphotransferase activity | Carbohydrate, D-ribose, nucleoside metabolic process; carbohydrate phosphorylation |
|  |  | Bradi1g07550 | mediator complex binding | pollen tube guidance |
|  |  | Bradi1g35600 | Tryptophan synthase activity; pyridoxal phosphate binding; L-serine hydro-lyase activity;oxidoreductase activity | Tryptophan biosynthetic and metabolic process |
|  |  | Bradi1g12040 | Catalytic activity | Unknown |
|  |  | Bradi3g54460 | SNARE, phospholipid, clathrin and clathrin heavy chain binding | Clathrin-dependent endocytosis; vesicle budding from membrane; clathrin coat assembly |
|  |  | Bradi2g42190 | Hydrolase activity | Cytokinin biosynthetic process; regulation of meristem development |
|  |  | Bradi1g47067 | Unknown | Unknown |
|  |  | Bradi2g56037 | Rhodanese | Oxidized Rhodanese |
|  |  | Bradi1g22850 | Aspartic-type endopeptidase, peptidase and hydrolase activity | Proteolysis; protein catabolic process |
| BdTPS5 | Bradi2g19710 | Bradi4g45180 | Phosphatidylinositol phospholipase C activity; hydrolase activity; phosphoric diester hydrolase activity | Lipid metabolic and catabolic process; signal transduction; release of sequestered calcium ion into cytosol |
|  |  | Bradi3g14620 | Protein kinase activity; calmodulin and ATP binding | protein phosphorylation; response to cold |
|  |  | Bradi3g43587 | Nucleotide and ATP binding; microtubule motor activity | Microtubule-based movement |
|  |  | Bradi1g18380 | Protein and phosphatidylinositol binding | Protein localization to cilium |
|  |  | Bradi5g15070 | Protein kinase activity; protein and ATP binding | Protein autophosphorylation; protein phosphorylation |
|  |  | Bradi1g69380 | Protein kinase activity | Mitotic cell cycle; synapsis; protein phosphorylation |
| BdTPS6 | Bradi2g49870 | Bradi2g56070 | Protein binding; phosphatidylinositol binding; F-box | Unknown |
|  |  | Bradi4g13280 | Unknown | Unknown |
|  |  | Bradi4g09597 | ADP binding | Disease resistance protein signature |
|  |  | Bradi5g18820 | Protein binding | Unknown |
|  |  | Bradi2g38510 | Protein kinase activity; ATP binding | Protein phosphorylation |
|  |  | Bradi1g68580 | PHD_Oberon | Cell fate specification; metal ion homeostasis; embryonic meristem initiation; embryonic pattern specification |
|  |  | Bradi2g59137 | DNA and protein binding | Cell fate specification; metal ion homeostasis; embryonic meristem initiation; embryonic pattern specification |
|  |  | Bradi1g20390 | Nucleotide, protein and ATP binding; protein kinase activity | Ethylene-responsive |
|  |  | Bradi3g59510 | Protein kinase activity; ATP binding | Unknown |
|  |  | Bradi1g53490 | Solute_trans_a; Fumarate reductase respiratory complex transmembrane subunits | Organic solute transporter Ostalpha |
|  |  | Bradi1g70920 | Nucleotide , calmodulin and ATP binding; calcium-transporting ATPase activity; hydrolase activity | Calcium ion transport; ATP hydrolysis coupled cation transmembrane transport |
| BdTPS7 | Bradi4g41580 | Bradi4g04360 | RNA binding; protein binding; zf-C3H1 | Unknown |
|  |  | Bradi1g44490 | Myb_DNA-bind_4 | Unknown |
|  |  | Bradi1g68870 | Protein binding | RNA modification |
|  |  | Bradi2g48320 | Sequence-specific DNA binding; transcription regulatory region DNA binding; Myb_DNA-bind_4 | Unknown |
|  |  | Bradi1g21290 | Cytochrome-c oxidase activity; Warburg's respiratory enzyme; Cytochrome C oxidase subunit h; COX6B | Cytochrome c oxidase |
| BdTPS9 | Bradi4g29730 | Bradi4g08640 | Unknown | Unknown |
|  |  | Bradi1g76170 | Catalytic activity; transcription cofactor activity; SAGA complex; Spt20 | Histone acetyltransferase etc |
|  |  | Bradi1g06200 | Iron ion binding; oxidoreductase and electron transfer activity; FAD binding | Electron transport chain; oxidation-reduction process |
|  |  | Bradi4g33370 | DNA binding; DNA-binding transcription factor activity; sequence-specific DNA binding | Regulation of transcription, DNA-templated |
|  |  | Bradi1g64920 | Protein phosphatase inhibitor activity; abscisic acid binding; signaling receptor activity | Defense response; response to biotic stimulus; abscisic acid-activated signaling pathway |
|  |  | Bradi3g23167 | Transcription cofactor activity; SAGA complex; Spt20 | Histone acetyltransferase etc |
|  |  | Bradi3g35067 | Oxidoreductase activity; flavin adenine dinucleotide binding; FAD binding | Oxidation-reduction process |
|  |  | Bradi3g03150 | Ataxin-2 C-terminal region | Unknown |
|  |  | Bradi3g20740 | Unknown | Unknown |
|  |  | Bradi3g15660 | Nucleotide, signaling receptor and ATP binding; protein kinase, transferase and protein homodimerization activity | Protein phosphorylation; brassinosteroid mediated signaling pathway; detection of brassinosteroid stimulus |
|  |  | Bradi5g17880 | Transferase activity, transferring glycosyl groups | Protein glycosylation |
|  |  | Bradi4g12770 | ADP binding | Disease resistance protein signature |
|  |  | Bradi1g05660 | Nucleic acid and zinc ion binding; nuclease, exonuclease and hydrolase activity | Nucleobase-containing compound metabolic process; RNA phosphodiester bond hydrolysis, exonucleolytic; mRNA processing |
|  |  | Bradi1g29560 | ADP binding; Ribonuclease Inhibitor | Disease resistance protein signature |
| BdTPPB | Bradi1g27470 | Bradi1g57670 | Protein kinase activity; ATP binding; protein phosphorylation; carbohydrate binding | Phosphate etc |
|  |  | Bradi4g01680 | Protein kinase activity; protein binding; ATP binding | Phosphate etc |
|  |  | Bradi3g28137 | Cellular_component | Hypersensitive-induced response protein 1 |
|  |  | Bradi5g11020 | LURP-one-related | Unknown |
| BdTPPC | Bradi3g50810 | Bradi1g68210 | Unknown | Senescence-associated protein |
|  |  | Bradi4g09210 | Serine-type endopeptidase activity; peptidase activity; hydrolase activity | Proteolysis |
|  |  | Bradi1g16480 | Nucleotide binding | Protein phosphorylation |
|  |  | Bradi3g43390 | Aspartic-type endopeptidase activity; peptidase activity; hydrolase activity | Protein catabolic process; proteolysis |
|  |  | Bradi2g39220 | Ubiquitin protein ligase activity | Protein ubiquitination |
|  |  | Bradi2g42190 | Hydrolase activity, hydrolyzing N-glycosyl compounds | Cytokinin biosynthetic process; regulation of meristem development |
|  |  | Bradi3g59690 | Unknown | Unknown |
| BdTPPD | Bradi5g17890 | Bradi1g57540 | Cysteine-rich secretory protein family | Pathogenesis-related |
| BdTPPE | Bradi3g35590 | Bradi1g17450 | Ricin-type beta-trefoil lectin domain-like | Unknown |
|  |  | Bradi1g73170 | Maltose, sucrose:proton symporter activity; sucrose and salicin transmembrane transporter activity | Pollen germination; sucrose transport; salicin transport |
| BdTPPF | Bradi4g29030 | Bradi1g49570 | DNA-binding transcription factor activity | Regulation of transcription, DNA-templated |
|  |  | Bradi5g08650 | Tify domain profile; jasmonate ZIM domain-containing protein | Response to wounding; regulation of defense response; regulation of jasmonic acid mediated signaling pathway |
|  |  | Bradi3g29620 | Unknown | Late embryogenesis abundant protein |
|  |  | Bradi3g38140 | DNA binding; AP2_ERF | Ethylene responsive element binding protein signature |
|  |  | Bradi5g11970 | Unknown | Unknown |
|  |  | Bradi1g72610 | Tify domain profile; jasmonate ZIM domain-containing protein | Response to wounding; regulation of defense response; regulation of jasmonic acid mediated signaling pathway |
|  |  | Bradi1g03770 | Nucleic acid binding; metal ion binding; C2H2-type zinc finger | Interacting selectively and non-covalently with any nucleic acid and metal ion |
|  |  | Bradi3g23190 | Tify domain profile; jasmonate ZIM domain-containing protein | Response to wounding; regulation of defense response; regulation of jasmonic acid mediated signaling pathway |
| BdTPPG | Bradi3g58960 | Bradi1g07390 | Lzipper-MIP1 | Unknown |
| BdTPPH | Bradi1g60950 | Bradi3g06800 | Unknown | Unknown |
|  |  | Bradi3g16070 | Unknown | Unknown |
|  |  | Bradi1g20350 | Unknown | Unknown |
|  |  | Bradi2g15650 | Unknown | Unknown |
| BdTPPI | Bradi1g45480 | Bradi5g00940 | Protein kinase activity; ATP binding | Protein phosphorylation |
|  |  | Bradi2g15440 | Galactosyltransferase activity | Protein glycosylation |
|  |  |  |  |  |
