# Supplementary figures and images for: Genome-Wide Identification, Evolution, and Expression Analysis of TPS and TPP Gene Families in Brachypodium distachyon
Source: Plants (Basel). 2019 Sep 23;8(10):362. doi: 10.3390/plants8100362 (PMC6843561; doi:10.3390/plants8100362)

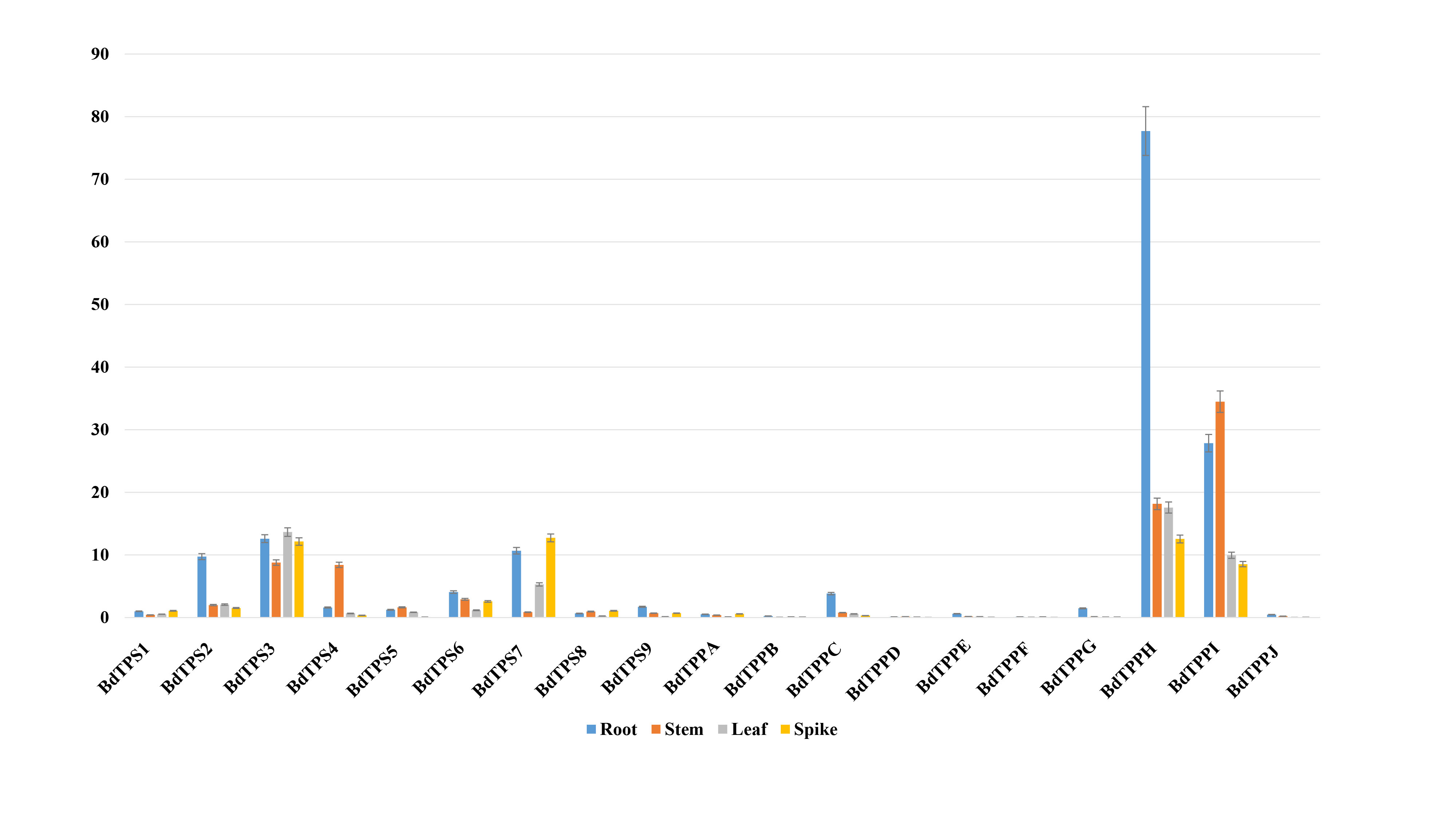

Supplement: Supplementary file 1 [file plants-08-00362-s001.zip › Figure S1.jpg]

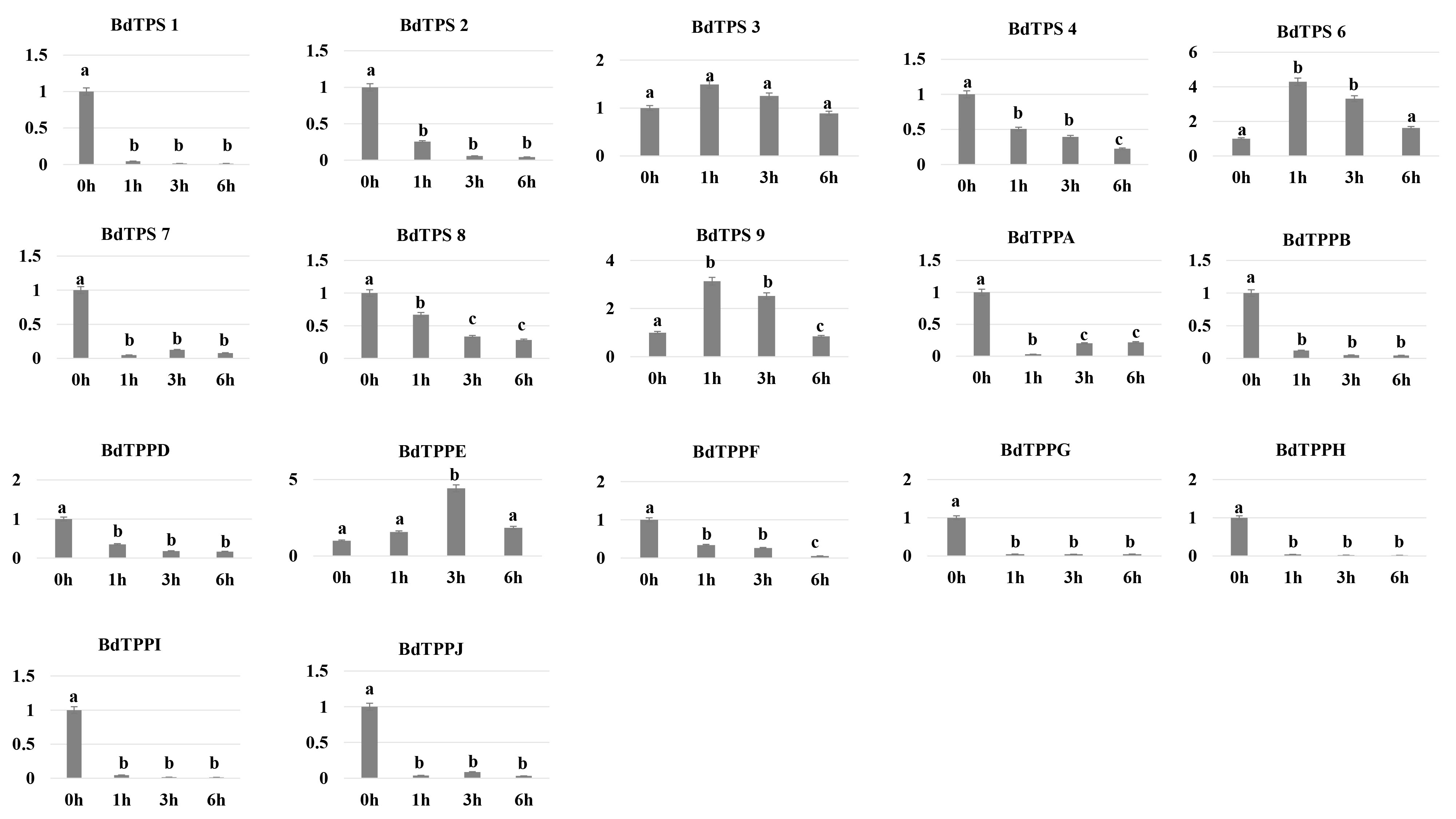

Supplement: Supplementary file 1 [file plants-08-00362-s001.zip › Figure S2.jpg]

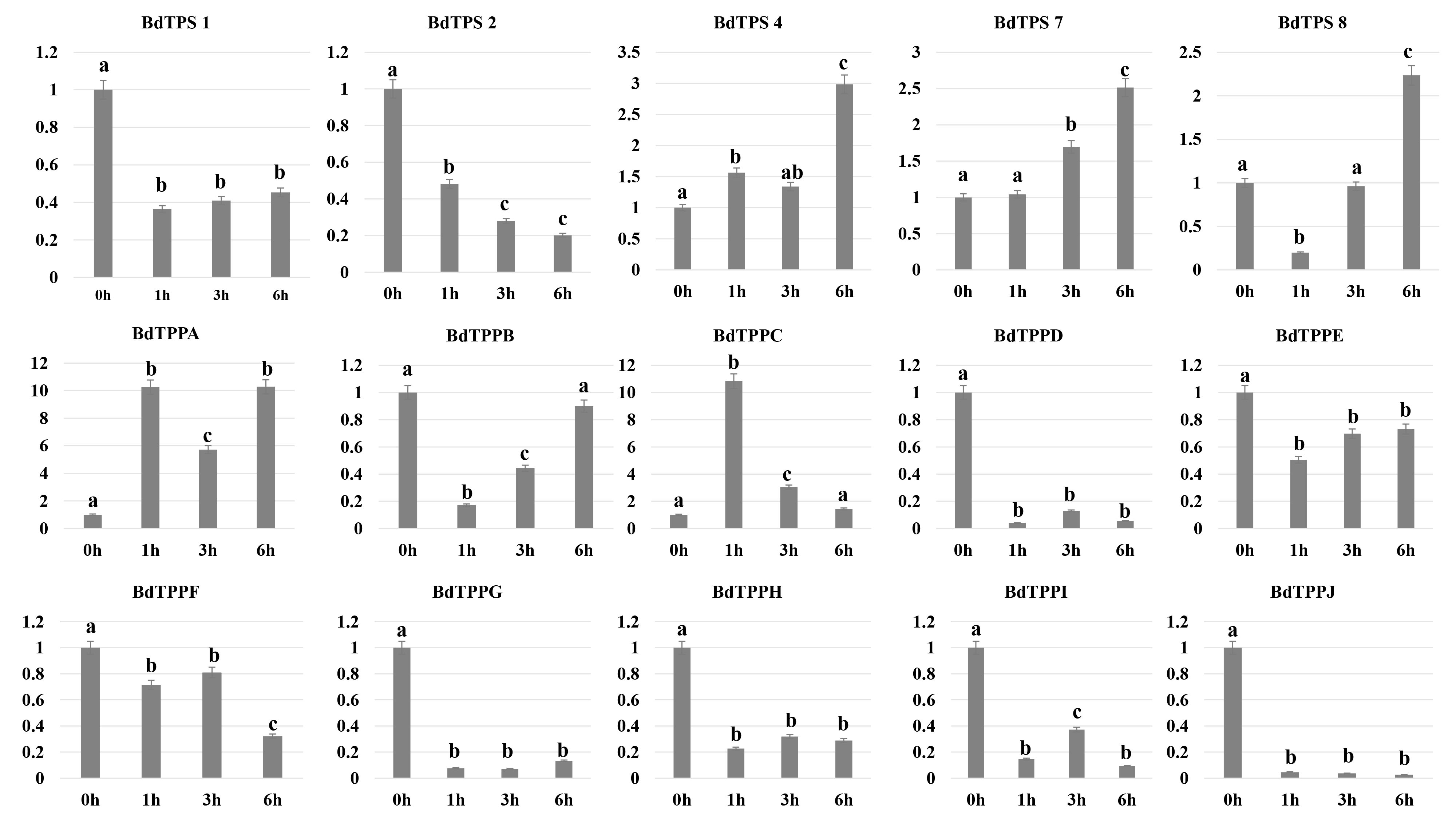

Supplement: Supplementary file 1 [file plants-08-00362-s001.zip › Figure S3.jpg]

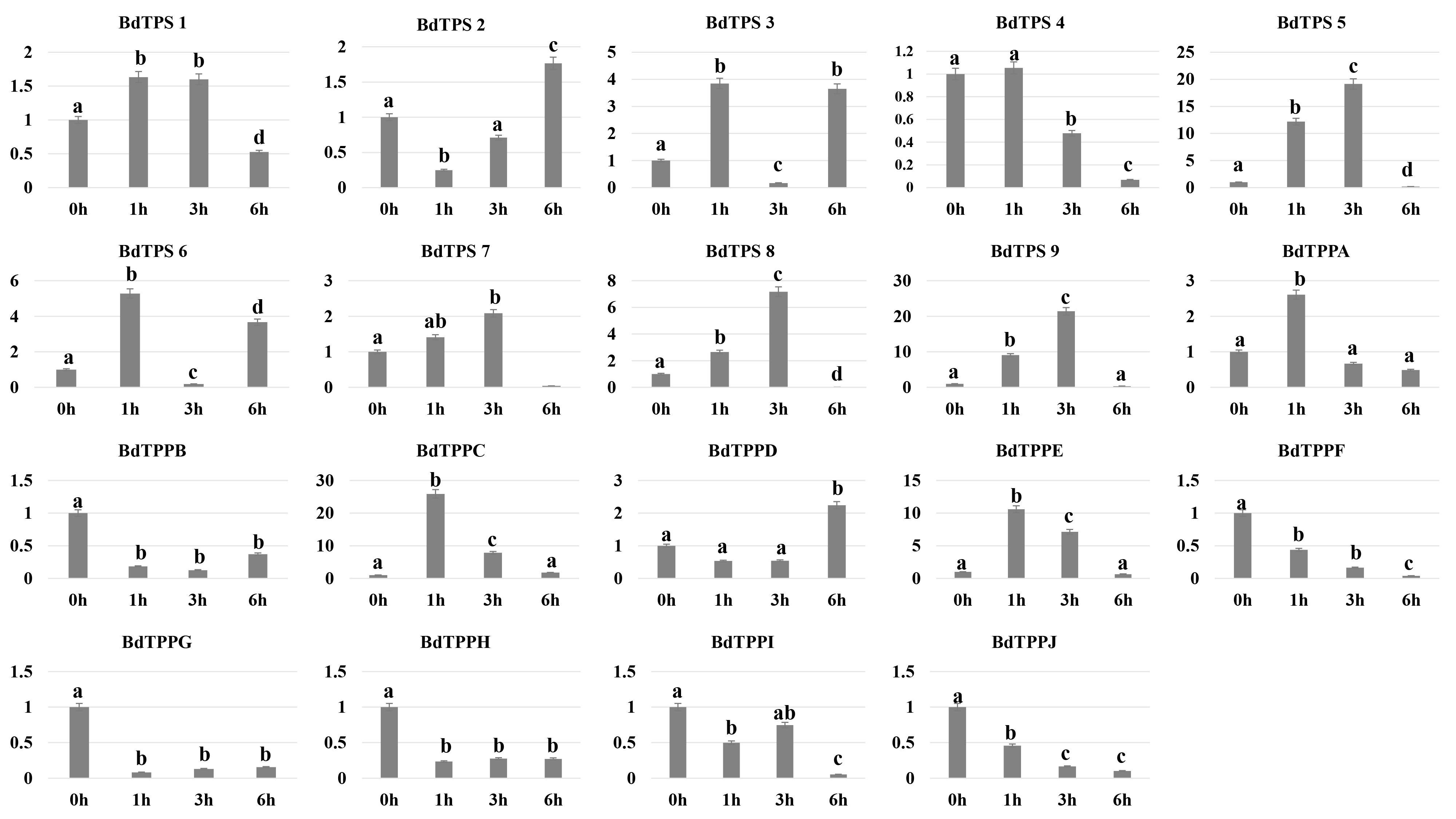

Supplement: Supplementary file 1 [file plants-08-00362-s001.zip › Figure S4.jpg]

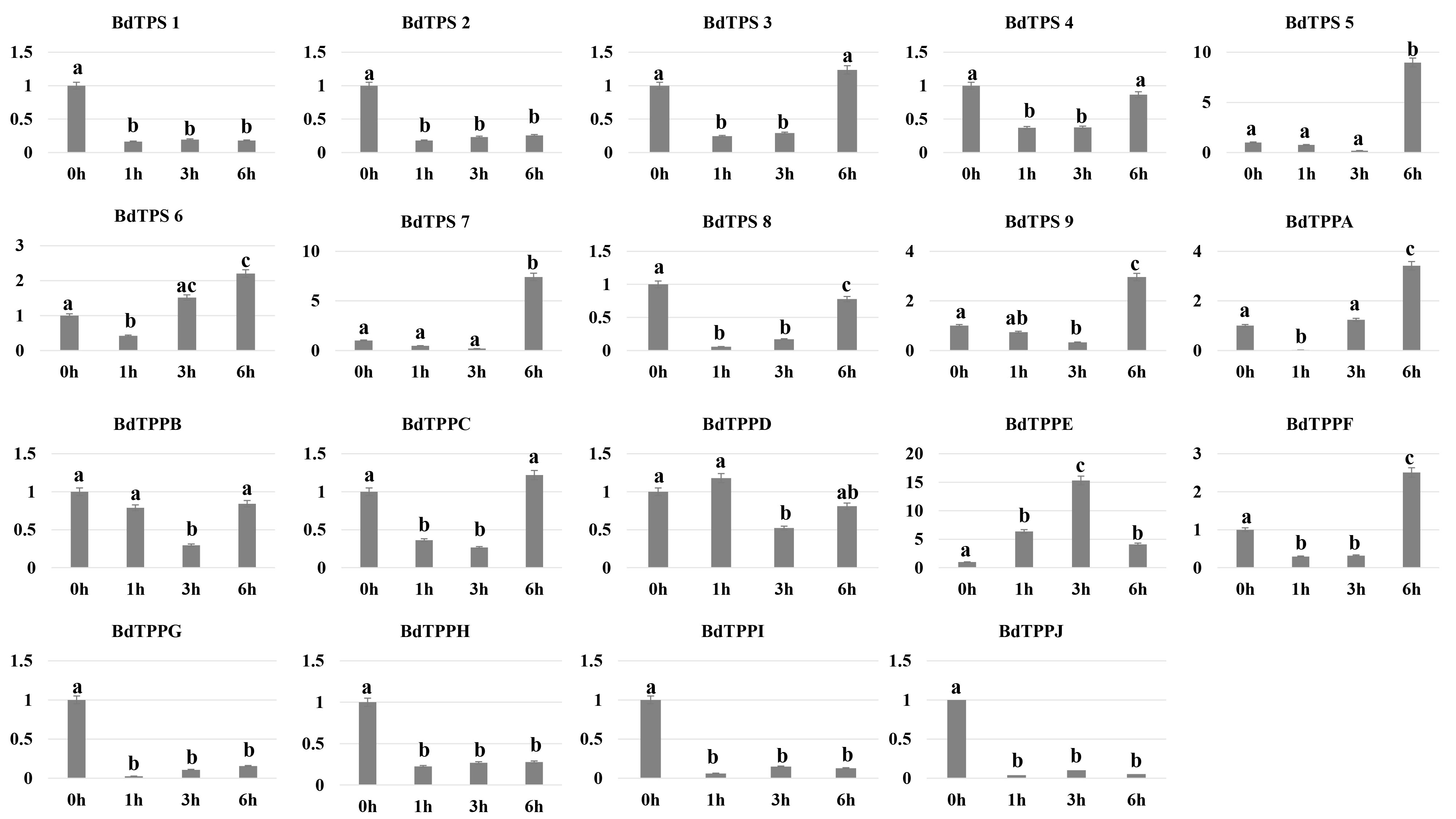

Supplement: Supplementary file 1 [file plants-08-00362-s001.zip › Figure S5.jpg]
